# FluNexus: A versatile web platform for antigenic prediction and visualization of influenza A viruses

**DOI:** 10.1002/imt2.70127

**Published:** 2026-05-02

**Authors:** Xingyi Li, Chunyan Zhou, Han Wu, Kexin Xiao, Jun Hao, Dongmin Zhao, Guohua Deng, Yue Li, Jia Gu, Weigang Cai, Junnan Zhu, Jiajie Peng, Min Li, Yan Liu, Xuequn Shang, Hualan Chen, Huihui Kong

**Affiliations:** ^1^ School of Computer Science Northwestern Polytechnical University Xi'an Shaanxi China; ^2^ State Key Laboratory of Animal Disease Control and Prevention, Harbin Veterinary Research Institute Chinese Academy of Agricultural Sciences Harbin China; ^3^ Changchun Veterinary Research Institute Chinese Academy of Agricultural Sciences Changchun China; ^4^ Faculty of Data Science City University of Macau Macau China; ^5^ Worldwide Influenza Centre The Francis Crick Institute London UK; ^6^ State Key Laboratory of Multimodal Artificial Intelligence Systems, Institute of Automation Chinese Academy of Sciences Beijing China; ^7^ School of Computer Science and Engineering Central South University Changsha China

## Abstract

FluNexus is a versatile platform for the antigenic prediction and visualization of influenza A viruses, including: (i) Online data preprocessing module. (ii) Online antigenic prediction module. (iii) Visualization module for mapping antigenic evolution.

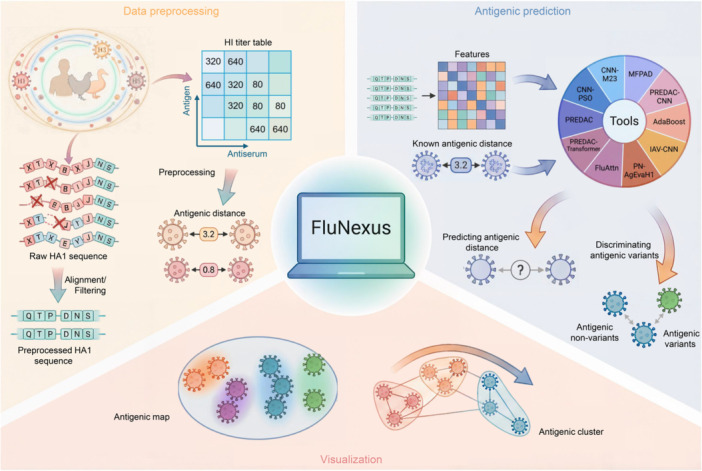

To the Editor,

Influenza A viruses pose a major global health threat due to their high mutation rates, broad host ranges, and pandemic potential [1]. Human seasonal influenza is primarily driven by H1 and H3 subtypes, which cause 3–5 million severe cases and 290,000–650,000 respiratory‐related deaths annually worldwide. Beyond seasonal outbreaks, avian influenza viruses also present a pandemic threat, particularly the H5 subtype, whose widespread circulation in birds caused serious damage to poultry production, the dairy industry, and public human health [2]. Vaccination is a cornerstone strategy for controlling both seasonal and avian influenza. However, the protective efficacy of vaccines is commonly undermined by rapid antigenic evolution, driven predominantly by mutations in the hemagglutinin subunit 1 (HA1) proteins that confer immune escape. Timely deployment of antigenically matched vaccines can effectively control or even eliminate circulating viruses, as demonstrated by the control of H7N9 in poultry [3]. However, delays in updating vaccines significantly increase outbreak risks, underscoring an urgent need for rapid, high‐throughput, and accurate methods to assess viral antigenicity.

Conventionally, viral antigenicity is assessed using serological assays such as hemagglutination inhibition (HI), which are labor‐intensive, time‐consuming, and low‐throughput. Advances in sequencing technologies and the resulting accumulation of viral genomic data have enabled the development of sequence‐based computational methods for rapid antigenic prediction. For example, Smith et al. [4] depict the antigenic evolution of A/H3N2 using modified metric multidimensional scaling to reveal cluster‐wise, low‐dimensional shifts closely aligned with genetic changes, which are named as Racmacs. This methodology has been extensively refined in follow‐up studies [5, 6]. Subsequent machine learning [7, 8, 9, 10] and deep learning‐based methods [11, 12, 13, 14, 15, 16] have further improved antigenic predictions by integrating HI and large‐scale sequencing data. Nevertheless, user‐friendly tools for online data preprocessing, antigenic prediction, and visualization are still lacking. Most current methods require programming expertise, creating a barrier for bench scientists without computational backgrounds. Consequently, there is an urgent need for a versatile, online platform to systematically monitor viral antigenic evolution and inform timely vaccine updates.

To address these limitations, we propose FluNexus, a versatile web platform for antigenic prediction and visualization of influenza A viruses with a user‐friendly interface. It first features interactive data preprocessing modules for HI and HA1 data. Next, FluNexus supports 10 state‐of‐the‐art sequence‐based computational algorithms for online antigenic prediction and presents a comprehensive benchmarking framework to evaluate their performance for H1, H3, and H5 subtypes in terms of discriminating antigenic variants from non‐variants, predicting antigenic distances of virus pairs, and computational time complexity, thereby offering practical recommendations for users conducting influenza antigenic prediction. Moreover, FluNexus provides the visualization of antigenic maps and antigenic clusters of influenza A viruses, and proposes an optimized strategy for antigenic cartography.

## PIPELINE AND WEB PLATFORM OVERVIEW

**FIGURE 1:**
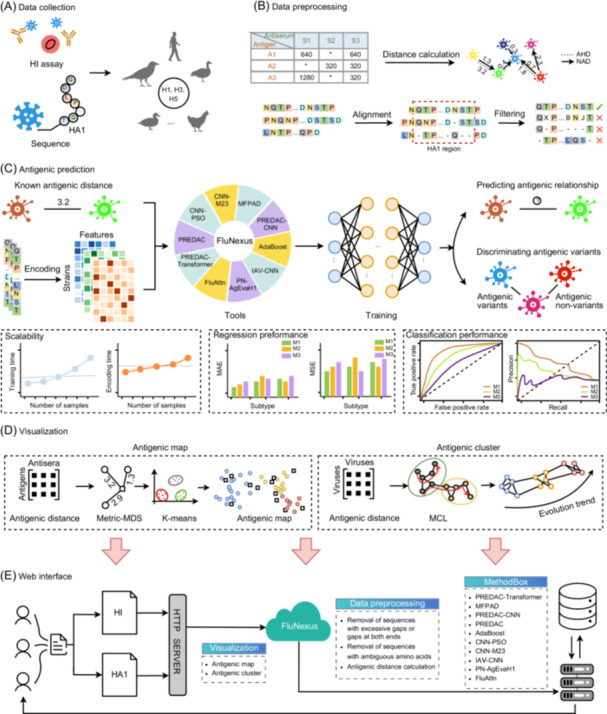
Overview of FluNexus. (A) Collection of hemagglutination inhibition (HI) titer data and hemagglutinin subunit 1 (HA1) protein sequences for H1, H3, and H5 subtypes. (B) Data preprocessing module for HA1 and HI data spanning the H1, H3, and H5 subtypes. (C) Antigenic prediction module for predicting antigenic distance and discriminating antigenic variants from non‐variants using accurate sequence‐based predictive methods. (D) Visualization module for mapping antigenic evolution of influenza A viruses, incorporating a novel manifold‐based method for antigenic cartography. (E) FluNexus empowers researchers with a versatile web platform for antigenic prediction and visualization of influenza A viruses.

FluNexus is a comprehensive web platform for the antigenic prediction and visualization of influenza A viruses, integrating three core components into a unified, accessible framework: (i) data preprocessing, (ii) online computational tools and evaluation, and (iii) antigenic visualization. The architecture and implementation of FluNexus are shown in Supplementary S1, and users can navigate easily among all functional modules through the web interface. A detailed user manual is available online at https://flunexus.com/tutorial.html, providing comprehensive guidance on data input, method usage, and result interpretation. The overall workflow is illustrated in Figure 1.

## DATA PREPROCESSING AND ANTIGENIC PREDICTION TOOLS

To facilitate convenient access to unified HA1 and HI data for antigenic prediction or visualization, FluNexus features a data preprocessing pipeline for users to process their own uploaded data on the “Data Preprocess” page. For HA1 sequences, FluNexus provides three quality control options: (i) remove uncertain amino acids (e.g., B, Z, J, X), (ii) remove sequences with gaps at both ends, and (iii) remove sequences with high gap ratios. For HI data, the platform facilitates the conversion of HI titers into antigenic distances. Specifically, antigenic distances of virus pairs can be calculated using either the Normalized Antigenic Distance (NAD) [4] or the log_2_‐transformed Archetti–Horsfall Distance (AHD) [7] (Supplementary S2). Based on the calculated antigenic distance, each virus pair can be classified as antigenically similar (distance < 2) or distinct (distance ≥ 2), consistent with criteria widely used in Centers for Disease Control and Prevention (CDC) or World Health Organization (WHO) surveillance reports (https://www.cdc.gov/fluview/surveillance/2025-week-52.html) and influenza antigenic cartography studies [4, 8].

Meanwhile, FluNexus provides both online and open‐source implementations (https://github.com/xingyili/FluNexus-methodbox) of 10 sequence‐based tools for predicting antigenic distance and discriminating antigenic variants from non‐variants. The integrated tools comprise PREDAC [9], CNN‐M23 [10], CNN‐PSO [11], MFPAD [12], PREDAC‐CNN [8], AdaBoost [13], IAV‐CNN [14], PN‐AgEvaH1 [15], PREDAC‐Transformer [16], and FluAttn [17] (Supplementary S3). On the “Tools” page in FluNexus, users can perform online analyses by uploading HA1 sequences and specifying the desired computational tool, virus subtype, and types of antigenic distance.

## BENCHMARKING OF ANTIGENIC PREDICTION METHODS

To guide rational model selection tailored to specific subtypes and research inquiries, we present a comprehensive benchmark evaluating the practical strengths and limitations of the 10 antigenic prediction methods. All methods are evaluated within a unified framework using identical datasets to ensure unbiased comparisons (see Supplementary S4 and Figure S1 for details) and evaluation metrics. The benchmarking framework is developed based on three key criteria: (i) the ability to discriminate antigenic variants from non‐variants, measured by the Area Under the Receiver Operating Characteristic Curve (AUC) and Area Under the Precision‐Recall Curve (AUPRC), (ii) the accuracy in inferring antigenic distances between virus pairs, measured by the mean absolute error (MAE) and mean squared error (MSE), and (iii) computational efficiency, quantified by the time for HA1 sequence encoding and model training. To ensure robust evaluation, we employ both cross‐validation and retrospective data partitioning strategies (see Supplementary S5 for details).

In experiments of discriminating antigenic variants from non‐variants using NAD antigenic distance (Figure S2A), AdaBoost exhibits superior performance across H1, H3, and H5 subtypes under cross‐validation, while FluAttn, MFPAD, and AdaBoost maintain high predictive accuracy in the retrospective testing scenario. For AHD‐based classification, MFPAD, AdaBoost, and FluAttn prove superior under both the cross‐validation and retrospective testing strategies (Figure S3A). Notably, we observe a performance decline for nearly all models under retrospective testing, suggesting a constrained capacity to generalize to temporally distant or unseen viral strains.

Regarding antigenic distance inference between virus pairs based on NAD antigenic distance (Figure S2B), AdaBoost and CNN‐M23 consistently yield optimal performance across H1, H3, and H5 subtypes under cross‐validation. AdaBoost further exhibits exceptional efficacy in retrospective testing, especially for the H3 subtype. In terms of the AHD antigenic distance (Figure S3B), MFPAD demonstrates robust predictive accuracy, specifically for the H1 and H5 subtypes. It is also evident that retrospective testing results in increased prediction errors relative to cross‐validation across all subtypes, with the most significant increase occurring in the H5 subtype.

Computational efficiency is assessed by measuring HA1 sequence encoding and model training time at incrementally increasing data scales. Observing that the original one‐hot encoding in AdaBoost creates a dimensionality bottleneck, we implement a sparse vector representation, denoted AdaBoost (Sparse One‐Hot), to mitigate this overhead. All benchmarking is conducted using 32‐thread parallelization. As illustrated in Figures S2C and S4, MFPAD and PREDAC achieve the highest encoding efficiency, while MFPAD, PN‐AgEvaH1, and PREDAC demonstrate superior efficiency in model training time. Conversely, IAV‐CNN and FluAttn suffer from high encoding cost, attributed respectively to the computationally intensive trigram sliding‐window process and the requirement for dynamic feature mining. IAV‐CNN also presents the highest training computational cost owing to its dense matrix operations. Notably, for the H1 subtype (NAD distance), AdaBoost displays significantly elevated training times, highlighting its limited scalability when applied to datasets containing large numbers of unique antigens and antisera. Ultimately, we integrate these results into a comprehensive performance summary (Figures S2D and S5).

## IMPROVED MAPPING OF ANTIGENIC EVOLUTION AND VISUALIZATION

**FIGURE 2:**
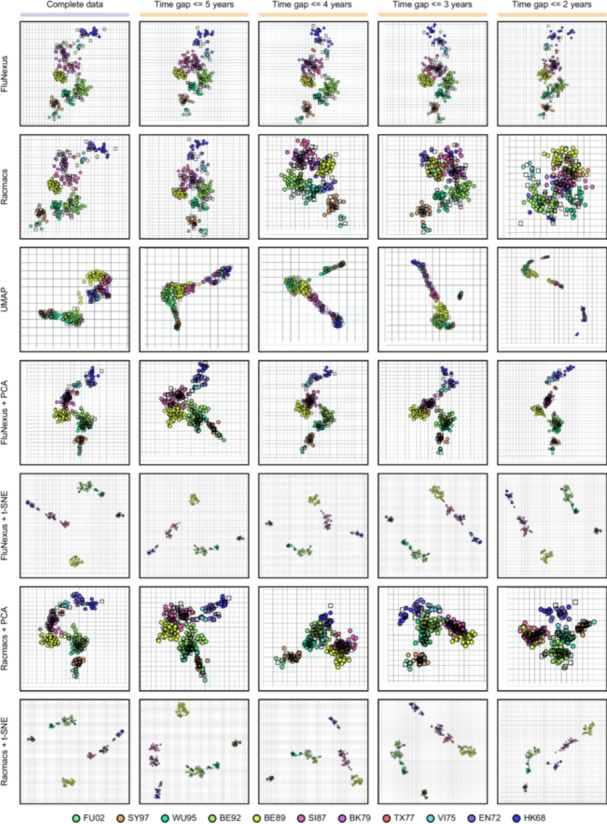
Antigenic maps generated by FluNexus, Racmacs, Uniform Manifold Approximation and Projection (UMAP), principal component analysis (PCA), and t‐distributed Stochastic Neighbor Embedding (t‐SNE) using the HI data of H3 subtype sourced from Smith et al. under temporal sampling. Antigens in maps are colored consistently with the Smith et al. study.

HI data are typically generated by testing a limited number of antisera against a panel of viral isolates, resulting in numerous missing antigen‐antiserum titers. This data sparsity poses challenges for the widely used antigenic visualization tool Racmacs, hindering the accurate depiction of antigenic relationships among large sets of viruses (Figure 2). To improve the accuracy of the antigenic map, we propose a novel manifold‐based algorithm (Supplementary S6) to more accurately position antigens and antisera, learning the underlying antigenic characterization in HI data. We employ the HI dataset to construct antigenic maps and conduct a comparative benchmark against Racmacs (https://github.com/acorg/Racmacs) [4], Uniform Manifold Approximation and Projection [18] (UMAP), principal component analysis (PCA) [19], and t‐distributed Stochastic Neighbor Embedding (t‐SNE) [20] (Supplementary S7).

To assess the capacity of FluNexus in preserving global evolutionary patterns under data‐limited conditions, we simulate data scarcity by progressively excluding HI measurements with antigen‐serum temporal intervals exceeding 5, 4, 3, and 2 years. This simulation strategy mirrors real‐world practices, where temporally proximate antigen‐serum pairs are typically prioritized in HI assays. FluNexus is benchmarked against established tools using the canonical H3 dataset from Smith et al. [4], a field‐recognized gold standard featuring a reference antigenic map. As shown in Figure 2, FluNexus demonstrates superior performance by preserving coherent antigenic evolutionary trajectories and exhibiting robustness under both complete and temporally restricted data conditions. Quantitatively, FluNexus achieves the highest concordance with true antigenic relationship (MAE = 0.64, MSE = 0.84 for complete HI titers, Supplementary S8, Table S1 and Table S2). This suggests that our approach robustly captures the antigenic relationships from high‐dimensional immunological data, effectively mitigating the noise associated with temporal sampling. Thus, the FluNexus‐derived antigenic maps can be reliably applied to detect antigenic variants and prioritize vaccine strains in response to rapid viral evolution.

We subsequently evaluate FluNexus using HI datasets curated from the annual and interim reports of the Worldwide Influenza Centre at the Francis Crick Institute. Unlike the dataset from Smith et al., this H3 evolutionary landscape lacks a unified, widely accepted expert‐curated standard. To ensure robust map identifiability in this context, we restrict the analysis to viruses that present as both test antigens and reference antisera. The results show that FluNexus generates stable antigenic maps that preserve interpretable global evolutionary patterns and remain robust against data sparsity induced by temporal sampling (Figure S6). Extended evaluation on H1 and H5 datasets also validates the subtype generalizability of FluNexus (Figures S7 and S8).

The FluNexus web platform incorporates two interactive visualization modules: the antigenic map and the antigenic cluster. Users can construct antigenic maps using both the proposed manifold‐based method and the established Racmacs method, with support for both HI data and NAD value inputs. An integrated control panel facilitates the refinement of the generated map, allowing for the adjustment of key parameters such as cartographic dimensionality (projecting antigens and antisera in high‐dimensional immunological data to 2D or 3D cartography), the number of clusters, the random seed, and the number of optimizers. Furthermore, point properties (e.g., size, shape, and color) remain dynamically adjustable post‐rendering to enable customized visualization. The antigenic cluster module in FluNexus is implemented in accordance with PREDAC [9] (see Supplementary S7 for details). The initial positions of the points are determined using a built‐in “spring” layout (see Supplementary S9 for details). Similar to the antigenic map, users can adjust the size, shape, and color of points, and manually refine the layout via drag‐and‐drop.

## CONCLUSION

In this study, we present FluNexus, a one‐stop‐shop web platform that streamlines antigenic prediction and visualization for influenza A viruses. FluNexus is a versatile platform that integrates interactive modules for data preprocessing, online antigenic prediction with practical guidance for researchers, and visualization of influenza A virus antigenic evolution. Specifically, FluNexus proposes a novel manifold‐based method for positioning antigens and antisera, ensuring the generation of more accurate antigenic cartographies, particularly when HI data is sparse.

FluNexus lowers the technical barrier, empowering researchers without specialized computational backgrounds to perform antigenic analysis of the influenza A virus. Notably, beyond its application to influenza A virus, the core workflow and visualization framework of FluNexus can also be applied to other pathogens when assay‐derived antigenic measurements and corresponding genomic sequences are available. Overall, FluNexus is poised to significantly aid virologists and public health officials in tracking antigenic evolution and improving vaccine strain selection.

## AUTHOR CONTRIBUTIONS

**Xingyi Li**: Conceptualization; funding acquisition; investigation; project administration; supervision; visualization; writing—review and editing. **Chunyan Zhou**: Data curation; methodology; software; writing—original draft. **Han Wu**: Data curation; methodology; software; writing—original draft. **Kexin Xiao**: Formal analysis; investigation; validation. **Jun Hao**: Formal analysis; validation; investigation. **Dongmin Zhao**: Formal analysis; validation; investigation. **Guohua Deng**: Data curation; formal analysis. **Yue Li**: Software; validation. **Jia Gu**: Software; validation. **Weigang Cai**: Data curation; formal analysis. **Junnan Zhu**: Formal analysis; validation; investigation. **Jiajie Peng**: Software; validation. **Min Li**: Writing—review and editing; validation; supervision; project administration. **Yan Liu**: Data curation; formal analysis; supervision; project administration. **Xuequn Shang**: Resources; methodology; funding acquisition; project administration. **Hualan Chen**: Data curation; resources; methodology; supervision; project administration. **Huihui Kong**: Data curation; formal analysis; methodology; supervision; funding acquisition; project administration; writing—review and editing. All authors have read the final manuscript and approved it for publication. All authors have read the final manuscript and approved it for publication.

## CONFLICT OF INTEREST STATEMENT

The authors declare no conflicts of interest.

## ETHICS STATEMENT

The ethical protocols were reviewed and approved by the Committee on the Ethics of Animal Experiments at the Harbin Veterinary Research Institute, Chinese Academy of Agricultural Sciences (250220‐04‐GJ).

## Supporting information


**Figure S1:** Radar plot showing the prevalence of MetS and its components in the overall population and stratified by sex.
**Figure S2:** Boxplots comparing long‐term exposure levels to PM_2.5_, NO2, and ambient temperature between MetS and non‐MetS participants.
**Figure S3:** Phylum and genus ‐level relative abundance of gut microbiota in non‐MetS and MetS groups, based on 16S rRNA gene sequencing.
**Figure S4:** Elbow plot for selecting the optimal number of gut microbial community clusters.
**Figure S5:** The process of long‐term exposure assessment to air pollutants.
**Figure S6:** Random forest variable‐importance ranking of six ambient air pollutants (PM_2.5_, NO_2_, CO, PM_10_, SO_2_, and O_3_) for predicting MetS.
**Figure S7:** SHAP‐based feature importance of six ambient air pollutants in the XGBoost model for predicting MetS.

**Table S1:** Prevalence of metabolic syndrome and its diagnostic components.
**Table S2:** General characteristics of study participants by MetS status.
**Table S3:** ORs and 95% CIs of the associations between environmental factors and MetS.
**Table S4:** Mediation of the associations between environmental exposures and MetS by selected gut microbial genera.
**Table S5:** Variables used in the spatiotemporal land use regression (LUR) model.
**Table S6:** The definitions of each category of included variables.

## Data Availability

HA1 sequences for H1 and H3 influenza viruses can be downloaded from GISAID at https://gisaid.org/. Corresponding HI data for H1 and H3 subtypes can be downloaded at https://www.crick.ac.uk/research/platforms-and-facilities/worldwide-influenza-centre/annual-and-interim-reports. Additional HA1 sequences and HI data of the H3 subtype can be downloaded from the study by Smith et al. [4]. HA1 sequences and H5‐specific HI data can be downloaded from GISAID and GitHub https://github.com/xingyili/FluNexus-methodbox/tree/main/H5_data, respectively. The source code implementing the ten antigenic prediction methods is available at https://github.com/xingyili/FluNexus-methodbox. Supporting materials (methods, figures, tables, graphical abstract, slides, videos, Chinese translated version, and updated materials) may be found in the online DOI or iMeta Science http://www.imeta.science/.
