# Supplementary material for: FluNexus: A versatile web platform for antigenic prediction and visualization of influenza A viruses
Source: Imeta. 2026 May 2;5(2):e70127. doi: 10.1002/imt2.70127 (PMC13147951; doi:10.1002/imt2.70127)
Supplement: Supplementary file 1 — Figure S1: Radar plot showing the prevalence of MetS and its components in the overall population and stratified by sex. Figure S2: Boxplots comparing long‐term exposure levels to PM2.5, NO2, and ambient temperature between MetS and non‐MetS participants. Figure S3: Phylum and genus ‐level relative abundance of gut microbiota in non‐MetS and MetS groups, based on 16S rRNA gene sequencing. Figure S4: Elbow plot for selecting the optimal number of gut microbial community clusters. Figure S5: The process of long‐term exposure assessment to air pollutants. Figure S6: Random forest variable‐importance ranking of six ambient air pollutants (PM2.5, NO2, CO, PM10, SO2, and O3) for predicting MetS. Figure S7: SHAP‐based feature importance of six ambient air pollutants in the XGBoost model for predicting MetS. [file IMT2-5-e70127-s002.docx]

# Supporting information to

# FluNexus: a versatile web platform for antigenic prediction and visualization of influenza A viruses

**Running title: FluNexus: antigenic prediction and visualization platform**

Xingyi Li^1^, Chunyan Zhou^1#^, Han Wu^1#^, Kexin Xiao^1^, Jun Hao^1^, Dongmin Zhao^1^, Guohua Deng^2^, Yue Li^3^, Jia Gu^4^, Weigang Cai^5^, Junnan Zhu^6^, Jiajie Peng^1^, Min Li^7^*, Yan Liu^3^*, Xuequn Shang^1^*, Hualan Chen^2^*, Huihui Kong^2^*

^1^School of Computer Science, Northwestern Polytechnical University, Xi’an, Shaanxi, 710072, China

^2^State Key Laboratory of Animal Disease Control and Prevention, Harbin Veterinary Research Institute, Chinese Academy of Agricultural Sciences, Harbin, 150069, China

^3^Changchun Veterinary Research Institute, Chinese Academy of Agricultural Sciences, Changchun, 130122, China

^4^Faculty of Data Science, City University of Macau, Macau, 999078, China

^5^Worldwide Influenza Centre, The Francis Crick Institute, London, NW1 1AT, UK

^6^State Key Laboratory of Multimodal Artificial Intelligence Systems, Institute of Automation, Chinese Academy of Sciences, Beijing, 100190, China

^7^School of Computer Science and Engineering, Central South University, Changsha, 410083, China

^#^These authors contributed equally: Chunyan Zhou, Han Wu

*Correspondence: konghuihui@caas.cn (Huihui Kong), chenhualan@caas.cn (Hualan Chen), shang@nwpu.edu.cn (Xuequn Shang), liu820512@163.com (Yan Liu), limin@mail.csu.edu.cn (Min Li)

## Supplementary Sections

### S1 Architecture and implementation of FluNexus

FluNexus adopts a decoupled architecture that separates the front-end and back-end components. The front end is responsible for rendering interactive interfaces and transmitting user requests to the back end, primarily implemented using HTML, CSS, and JavaScript. Antigenic map and antigenic cluster are visualized using Three.js (https://threejs.org/). Meanwhile, the back end orchestrates various computational tasks in response to user requests received from the front end and returns processed results to the client. It employs the Axum web framework (https://github.com/tokio-rs/axum) to handle HTTP request routing, processing, and managing WebSocket connections. In addition, FluNexus employs several strategies to safeguard user privacy. All data transmissions are secured via SSL certificates to ensure encrypted communication. No cookies are used for tracking or session management. Data submitted by the user resides only in memory for short-term processing and is never persisted on the server.

### S2 Data collection and preprocessing

We obtain all HA protein sequences of influenza A virus subtypes H1 and H3 from the GISAID database (https://gisaid.org/) [1], and additionally incorporate HA sequences of H3 subtype from Smith et al. [2]. For the H5 subtype, HA sequences are retrieved from GISAID and supplemented with additional sequences derived from virus isolates collected in China and processed in our BSL-3 laboratory. Sequences for each subtype are aligned separately using MAFFT, with A/California/04/2009 (H1N1) (isolate ID: EPI_ISL_376192), A/Aichi/2/1968 (H3N2) (isolate ID: EPI_ISL_123225), and A/Vietnam/1203/2004 (H5N1) (isolate ID: EPI_ISL_10656749), serving as reference strains for H1, H3, and H5, respectively. After alignment, only the HA1 subunit is retained, and the signal peptides (17, 16, and 16 amino acids for H1, H3, and H5 viruses, respectively) are removed. Sequences containing ambiguous amino acid codes (“B”, “Z”, “J”, or “X”) or with more than 10% gaps are excluded. Additionally, sequences with gaps at both ends are discarded, as these typically represent incomplete sequencing artifacts rather than true biological variations.

We collect HI titer data for H1 and H3 subtypes from 43 semiannual reports published by the Worldwide Influenza Centre at the Francis Crick Institute (https://www.crick.ac.uk/). Meanwhile, we further integrate HI measurements of H3 subtype for 79 vaccine strains and 253 reference viruses reported by Smith et al. [2] from 1968 to 2003. H5 subtype-specific HI titers are obtained from parallel HI assays performed in a BSL-3 laboratory, using representative Chinese H5 isolates and a serum panel of 27 samples covering 17 phylogenetic clades. The raw data are converted from a non-editable format into a standardized, unified data format to ensure compatibility with computational tools and analytical workflows. Meanwhile, the non-numeric titer values in the HI data are converted into numeric values following the procedure of Smith et al. [2].

The antigen-antiserum distances in the antigenic map corresponding to HI values are quantified using two measurements, NAD and AHD, introduced in previous studies [3−5]. Let $\text{H}_{\text{i}\text{,}\text{j}}$ denote the HI titer measured against antigen $\text{i}$ using antiserum from strain $\text{j}$. NAD can be defined as:

$$\text{D}_{\text{1}}\text{(}\text{i}\text{, }\text{j}\text{) = }\text{b}_{\text{j}}\text{-}\text{log}_{\text{2}} \text{(}\text{H}_{\text{i}\text{,}\text{j}}\text{)}$$

where $\text{b}_{\text{j}}$ represents the $\log_{2}$ of the maximum HI titer observed across all antigens for antiserum $\text{j}$. A difference of two or more units in NAD between two viruses generally indicates a significant antigenic drift [2], reflecting antigenic changes sufficient to reduce protective immunity and prompting vaccine updates. AHD is defined as:

$$\text{D}_{\text{2}}\text{(}\text{i}\text{,}\text{j}\text{) = }\text{log}_{\text{2}} \text{(}\sqrt{\frac{\text{H}_{\text{i}\text{,}\text{i}}\text{×}\text{H}_{\text{j}\text{,}\text{j}}}{\text{H}_{\text{i}\text{,}\text{j}}\text{×}\text{H}_{\text{j}\text{,}\text{i}}}}\text{)}$$

where $\text{H}_{\text{j}\text{,}\text{i}}$ denotes the HI titer measured using antiserum derived from strain $\text{i}$ against antigen $\text{j}$, $\text{H}_{\text{i}\text{,}\text{i}}$ denotes the homologous titer measured using antiserum from strain $\text{i}$ against its own antigen, and likewise for $\text{H}_{\text{j}\text{,}\text{j}}$. $\text{Log}_{\text{2}}$ transformation can compress the value distribution, alleviate data sparsity, and improve computational stability [6]. When the AHD distance between two viruses reaches or exceeds 2, they are considered antigenically distinct [6,7].

### S3 Settings of benchmarked methods

All machine learning methods are implemented following their studies. For deep learning-based methods, including CNN-PSO [3], CNN-M23 [8], PREDAC-CNN [7], IAV-CNN [9], PREDAC-Transformer [10], and FluAttn [11], we evaluate the performance of models on the test set using the optimal checkpoint corresponding to the lowest loss on the validation set across 1,000 training epochs.

The basic principles of the ten different methods are elucidated as follows:

- PREDAC [12]. PREDAC employs a Naive Bayes classifier to discriminate antigenic variants from non-variants by analyzing amino acid substitutions in HA1 sequences. It integrates diverse structural and physicochemical features, including mutations in epitopes, receptor-binding sites, and glycosylation substitutions, alongside shifts in physicochemical properties.
- CNN-PSO. CNN-PSO utilizes particle swarm optimization (PSO) to simultaneously select optimal physicochemical features and refine the architecture of a convolutional neural network (CNN), thereby achieving high-accuracy predictions of influenza virus antigenicity.
- CNN-M23. CNN-M23 encodes a HA1 sequences by applying PCA to the full AAindex, reducing amino acid representations to 11-dimensional vectors. This method transforms sequences into structured numerical matrices and employs an AlexNet-inspired CNN to predict pairwise antigenic distances, effectively capturing the complex mutation patterns driving antigenic drift.
- MFPAD [13]. MFPAD integrates multidimensional feature representations of HA1 sequences—comprising mutation counts, key antigenic sites, epitopes, glycosylation sites, and physicochemical properties—to train an XGBoost model for robust antigenicity prediction.
- PREDAC-CNN. PREDAC-CNN constructs subtype-specific feature dictionaries for H1N1 and H3N2 derived from physicochemical properties in the AAindex. By encoding HA1 sequences with these dictionaries, it utilizes a CNN to capture local amino acid interactions, demonstrating superior performance in variant identification.
- AdaBoost [14]. AdaBoost combines HA1 sequence data with associated metadata to predict antigenic distances. It leverages an adaptive boosting algorithm to learn a seasonally updated mapping from genetic variations to antigenic changes.
- IAV-CNN. IAV-CNN utilizes ProtVec to map amino acid 3-grams into 100-dimensional vectors, effectively capturing strain variability. It features a 2D CNN augmented with Squeeze-and-Excitation (SE) blocks to model channel interdependencies, thereby focusing the network's attention on residues critical for antigenicity.
- PN-AgEvaH1 [15]. PN-AgEvaH1 employs a random forest framework to evaluate antigenicity by integrating physicochemical properties with structure-derived correlations, accurately mapping sequence variations to antigenic characteristics.
- PREDAC-Transformer. PREDAC-Transformer leverages a transformer encoder with self-attention mechanisms to capture long-range sequence dependencies. It synergizes physicochemical properties with ESM-2 protein language model embeddings to enhance antigenic prediction capabilities.
- FluAttn. FluAttn introduces an attention-based feature mining framework to automatically identify and weight antigenicity-relevant properties from the AAindex. These adaptive features are combined with sequence differences to train a multilayer perceptron for precise antigenic prediction.

### S4 Characterization of benchmark datasets

The benchmark datasets are composed of paired HA1 sequences and HI titers. We focus on the HA1 region of the HA protein, as amino acid substitutions within this region—particularly those occurring at or near antigenic sites—play a pivotal role in driving immune escape and antigenic drift [2,16,17]. The HA1 datasets comprise 6727 sequences of 326 amino acids for H1 (1976–2024), 6353 sequences of 328 amino acids for H3 (1968–2024), and 77 sequences of 317 amino acids for H5 (1996–2020). Within these HA1 regions of each subtype, variable amino acid sites (Figure S1), defined as those with the most frequent residue in less than 75% of sequences, often correspond to known antigenic epitopes. Substitutions at these critical sites, such as sites 156, 164, and 186 in H1 [18−20]; 156 and 193 in H3 [21, 22]; and 185 and 189 in H5 [23,24], are recognized as primary drivers of antigenic drift.

HI data are quantitative measurements derived from the HI assay, which provides the assessments of antigenicity among influenza virus isolates within the same subtype. The assay involves serial dilution of serum samples followed by incubation with influenza viruses and red blood cells to measure the ability of antibodies to inhibit HA-mediated agglutination [25]. In this study, we compile comprehensive HI benchmark datasets for H1, H3, and H5 influenza A subtypes. H1 includes 6727 antigens and 79 antisera collected between 1976 and 2024, while H3 comprises 6329 antigens and 243 antisera spanning from 1968 to 2024. For the H5 subtype, the collection contains 77 antigens and 24 antisera covering the period from 1996 to 2020. Regarding data sources, HI data for the H1 subtype are exclusively retrieved from the annual and interim reports of the Worldwide Influenza Centre at the Francis Crick Institute. For the H3 subtype, we integrate data sourced from the Worldwide Influenza Centre at the Francis Crick Institute with the dataset from Smith et al. [2], which comprises 79 vaccine strains and 253 reference viruses spanning the period from 1968 to 2003. Meanwhile, we generate a comprehensive H5 subtype–specific HI dataset in our BSL-3 laboratory. This dataset spans most globally reported H5 antigenic groups, thereby bridging critical gaps in existing data resources. Specifically, antisera are raised in three specific-pathogen-free (SPF) chickens immunized with 0.5 mL of an inactivated vaccine virus carrying the indicated HA and NA genes, with six remaining internal genes derived from A/Puerto Rico/8/1934 (PR8), as described previously [26]. Sera from the three chickens are pooled and stored at −80 °C for subsequent antigenic analyses. HI assays are performed according to the WHO-recommended protocol [27].

### S5 Benchmark metrics and partitioning strategies for benchmarking analysis

In this study, we evaluate the performance of each method using the following metrics: MAE and MSE for quantifying antigenic distance between virus pairs, AUC and AUPRC for evaluating the ability to distinguish antigenic variants from non-variants. All metrics are first computed separately for each individual fold or test year, and the overall performance is calculated as the average of the per-fold or per-year results, weighted according to the number of test samples in each fold or year.

For the experiment of distinguishing antigenic variants from non-variants, labels are assigned to virus pairs based on their antigenic relationship: pairs exhibiting antigenic variation are designated as positive samples, whereas pairs that are antigenically similar are designated as negative samples. Subsequently, AUC is computed as the area under the Receiver Operating Characteristic curve, which plots the True Positive Rate (TPR) against the False Positive Rate (FPR) across all decision thresholds, and quantifies the model**'**s ability to discriminate between positive and negative virus pairs. Here,

$$\text{TPR}\text{ = }\frac{\text{TP}}{\text{TP}\text{ + }\text{FN}}$$

$$\text{FPR}\text{ = }\frac{\text{FP}}{\text{FP}\text{ + }\text{TN}}$$

where TP is the number of antigenically variant pairs correctly classified as variant, FP is the number of antigenically similar pairs incorrectly classified as variant, TN is the number of antigenically similar pairs correctly classified as similar, and FN is the number of antigenically variant pairs incorrectly classified as similar. AUPRC is computed as the area under the Precision-Recall curve, which plots Precision against Recall across different decision thresholds, and emphasizes performance on the positive class. Here,

$$\text{Precision}\text{ = }\frac{\text{TP}}{\text{TP}\text{ + }\text{FP}}$$

$$\text{Recall}\text{ = }\frac{\text{TP}}{\text{TP}\text{ + }\text{FN}}$$

For the antigenic distance prediction experiment, MAE and MSE can be calculated as follows:

$$\text{MAE}\text{ = }\frac{\text{1}}{\text{n}}\sum_{\text{i}\text{=1}}^{\text{n}} \text{|}\text{y}_{\text{i}}\text{-}{\hat{\text{y}}}_{\text{i}}\text{|}$$

$$\text{MSE}\text{ = }\frac{\text{1}}{\text{n}}\sum_{\text{i}\text{=1}}^{\text{n}} \text{(}\text{y}_{\text{i}}\text{-}{\hat{\text{y}}}_{\text{i}}\text{)}^{\text{2}}$$

where $\text{y}_{\text{i}}$ denotes the true antigenic distance between the $\text{i}$-th pair of virus strains, ${\hat{\text{y}}}_{\text{i}}$ is the corresponding value predicted by the model, and $\text{n}$ is the total number of virus pairs (i.e., the total number of samples).

Finally, the overall performance across all folds or test years is obtained by combining the per-fold or per-year results according to the number of test samples in each fold or year. Specifically, if $\text{M}_{\text{y}}$ denotes the metric for fold $\text{y}$ or year $\text{y}$, the overall metric is given by:

$$\text{M}_{\text{average}}\text{ }\text{= }\frac{\sum_{\text{y}\text{ = 1}}^{\text{Y}} \text{N}_{\text{y}}\text{ }\text{M}_{\text{y}}}{\sum_{\text{y}\text{ = 1}}^{\text{Y}} \text{N}_{\text{y}}}$$

where $\text{Y}$ is the number of folds or test years, and $\text{N}_{\text{y}}$ denotes the number of virus pairs in the test set for fold $\text{y}$ or year $\text{y}$.

During model evaluation, two data partitioning strategies are employed: cross-validation and retrospective testing. Cross-validation is conducted at the titer level using five-fold partitioning repeated ten times. For the retrospective testing, strains are partitioned according to their isolation year. Data collected prior to the target year are used for training and validation, while strains from the target year and the following year are reserved for testing. When a separate validation set is used, samples from the two years preceding the target year are designated for validation, and these validation samples are excluded from the training set. The target years for each dataset are listed below:

- NAD (H1 subtype): 2019, 2020, 2021, 2022, 2023
- NAD (H3 subtype): 2019, 2020, 2021, 2022, 2023
- NAD (H5 subtype): 2015, 2016, 2017, 2018, 2019
- AHD (H1 subtype): 2018, 2019, 2020, 2021, 2022
- AHD (H3 subtype): 2019, 2020, 2021, 2022, 2023
- AHD (H5 subtype): 2015, 2016, 2017, 2018, 2019

### S6 Improved antigenic map

The shape space theory [28] shows that antigen-antiserum distance metrics can be transformed into meaningful spatial representations, allowing antigenic relationships to be visualized in the form of antigenic maps. Smith et al. [2] have employed MDS, based on the NAD values, to embed both antigens and antisera into a low-dimensional Euclidean space, thereby enabling the construction of an antigenic map. However, the NAD matrix is characteristically sparse. This sparsity is inherent to the temporal bias [29] and can introduce the unrealistic positioning of antigenically distant strains in close proximity on the antigenic map.

Manifold-based methods offer an effective way to capture the intrinsic geometry of complicated data, addressing limitations of linear methods such as MDS when Euclidean distances fail to reflect true relationships. By incorporating geodesic information that reflects the underlying manifold structure, Isomap [30] successfully uses locally reliable measurements to recover the global structure of the underlying data space. Consequently, inspired by Isomap, we introduce geodesic distance to estimate the distance for antigen–antigen, antiserum–antiserum, and antigen–antiserum pairs that lack HI measurements. We first construct a weighted antigen–antiserum graph where the nodes represent the antigens or antisera and the edges are weighted by the NAD distance. This initial graph is then refined into a neighborhood graph by connecting each antigen or antiserum to its $\text{k}$ nearest neighbors. Geodesic distances on this refined graph are then calculated using Dijkstra**'**s algorithm. Then, low-dimensional embedding will be constructed based on the NAD and geodesic distances and the Limited-memory Broyden–Fletcher–Goldfarb–Shanno algorithm [31] is adopted to optimize the low-dimensional coordinates of antigens and antisera. Therefore, FluNexus can best preserve the manifold**'**s estimated intrinsic geometry and ensure the generation of more accurate antigenic cartographies.

Firstly, we represent each antigen and antiserum by a coordinate vector in a low-dimensional embedding space (e.g., 2D or 3D). Subsequently, let $\text{D}\text{ ∈ }\text{R}^{\text{M}\text{×}\text{N}}$ denote the NAD matrix, where $\text{M}$ and $\text{N}$ represent the number of antigens and antisera, respectively. We then construct an undirected weighted graph $\text{G}\text{ }\text{= (}\text{V}\text{,}\text{E}\text{,}\text{w}\text{)}$, where the node set $\text{V}$ contains all antigens and antisera, i.e., $\text{V}\text{ }\text{= \{}\text{v}_{\text{1}}\text{,…,}\text{v}_{\text{M}\text{+}\text{N}}\text{\}}$. The first $\text{M}$ nodes correspond to antigens, and the remaining $\text{N}$ nodes correspond to antisera. An edge $\text{(}\text{v}_{\text{i}}\text{,}\text{v}_{\text{M}\text{+}\text{j}}\text{)∈}\text{E}$ exists only if the NAD $\text{D}_{\text{ij}}$ is available, and the associated edge weight is assigned as $\text{w}\text{(}\text{v}_{\text{i}}\text{,}\text{v}_{\text{M}\text{+}\text{j}}\text{) = }\text{D}_{\text{ij}}$. To better preserve the local manifold structure, a K-Nearest Neighbors (KNN) graph is constructed by retaining undirected edges only between each node and its $\text{k}$ nearest neighbors based on the edge weights, and then the geodesic distances between all pairs of nodes are calculated using Dijkstra**'**s algorithm. This results in a symmetric geodesic distance matrix $\text{Δ ∈ }\text{R}^{\text{(}\text{M}\text{+}\text{N}\text{)×(}\text{M}\text{+}\text{N}\text{)}}$, where each entry $\text{Δ}_{\text{ij}}$ denotes the length of the shortest path between node $\text{v}_{\text{i}}$ and node $\text{v}_{\text{j}}$ in the KNN graph. Subsequently, in order to flexibly modulate the influence of geodesic distances in the embedding process, we apply an affine transformation to the original geodesic distances $\text{Δ}_{\text{ij}}$:

$${\hat{\text{Δ}}}_{\text{ij}}\text{ }\text{= }\text{α}\text{⋅}\text{Δ}_{\text{ij}}\text{ }\text{+ }\text{β}$$

where $\text{α}$ and $\text{β}$ are hyperparameters that control the scaling and offset of the geodesic distances. Based on the KNN graph and transformed geodesic distances, we define four disjoint sets of node pairs:

- $\text{P}_{\text{obs}}$: the set of antigen–antiserum pairs with NAD measurements.
- $\text{P}_{\text{ag-sr}}$: the set of antigen–antiserum pairs without NAD measurements. Pairs with geodesic distance ${\hat{\text{Δ}}}_{\text{ij}}\text{ }\text{< }\text{r}$ are excluded.
- $\text{P}_{\text{ag-ag}}$: the set of antigen–antigen pairs. Pairs with geodesic distance ${\hat{\text{Δ}}}_{\text{ij}}\text{ }\text{< }\text{r}$ are excluded.
- $\text{P}_{\text{sr-sr}}$: the set of antiserum–antiserum pairs. Pairs with geodesic distance ${\hat{\text{Δ}}}_{\text{ij}}\text{ }\text{< }\text{r}$ are excluded.

Here, $\text{r}\text{>0}$ is a hyperparameter representing the minimum geodesic radius that excludes pairs with small geodesic distance in the high-dimensional graph. Subsequently, before defining the loss terms, we introduce a gating mechanism to softly activate penalties only when certain distance constraints are violated. Here,

$$\text{σ}\text{(}\text{x}\text{) =}\frac{\text{1}}{\text{1+}\text{exp}^{\text{-}\text{ηx}}}\text{ }$$

is a sigmoid function that smoothly activates the error only when $\text{x}\text{ > 0}$, with $\text{η}\text{ > 0}$ controlling the sharpness of the transition. With the above definitions, the optimization objective comprises the following four terms, designed to learn low-dimensional representations for all antigens and antisera such that the distances in the embedded space reflect the original NADs where available, and approximate the underlying manifold structure and the geodesic structure where direct measurements are missing.

1. For pairs $\text{(}\text{i}\text{, }\text{j}\text{) ∈ }\text{P}_{\text{obs}}$, we define the residual $\text{ξ}_{\text{ij}}\text{ }\text{= }\text{D}_{\text{ij}}\text{ }\text{- }\text{d}_{\text{ij}}$, where $\text{d}_{\text{ij}}$ denotes the Euclidean distance between nodes $\text{v}_{\text{i}}$ and $\text{v}_{\text{j}}$ in the low-dimensional embedding space, and minimize the squared difference between embedded and observed distances:

$$\text{e}_{\text{obs}}\text{ }\text{= }\sum_{\text{(i,j)}\text{ }\text{∈}\text{ }\text{P}_{\text{obs}}} \left\{ \begin{matrix} \text{σ(}\text{ξ}_{\text{ij}}\text{)⋅(}\text{ξ}_{\text{ij}}\text{)}^{\text{2}}\text{,} & \text{if (i, j) ∈ }\text{P}_{\text{obs}}^{\text{thr}} \\ \left( \text{ξ}_{\text{ij}} \right)^{\text{2}}\text{,} & \text{otherwise} \end{matrix} \right.$$

The subset $\text{P}\text{obs}^{\text{thr}}\text{ }\text{⊂ }\text{P}\text{obs}$ contains antigen–antiserum pairs whose HI measurements are thresholded values, typically reported as being below a detection limit (e.g., < 10).

1. For pairs $\text{(}\text{i}\text{, }\text{j}\text{) ∈ \{}\text{P}_{\text{ag}\text{-}\text{sr}}\text{, }\text{P}_{\text{ag}\text{-}\text{ag}}\text{, }\text{P}_{\text{sr}\text{-}\text{sr}}\text{\}}$, we introduce a soft constraint that adds a penalty to the objective function only when $\text{d}_{\text{ij}}\text{ }\text{< }{\tilde{\text{Δ}}}_{\text{ij}}$, i.e., when the distance between the node pair is smaller than their corresponding geodesic distance:

$$\text{e}_{\text{k}}\text{ }\text{=}\text{ }\sum_{\text{(i,j)}\text{ }\text{∈}\text{ }\text{P}_{\text{k}}} \text{σ}\text{(}{\hat{\text{Δ}}}_{\text{i,j}}\text{ }\text{-}\text{ }\text{d}_{\text{ij}}\text{)⋅(}{\hat{\text{Δ}}}_{\text{i,j}}\text{ }\text{-}\text{ }\text{d}_{\text{ij}}\text{)}^{\text{2}}\text{k}\text{ }\text{∈}\text{ }\text{\{ag-sr, ag-ag, sr-sr\}}$$

Finally, the total objective function is a weighted sum:

$$\text{e}\text{ = }\text{e}_{\text{obs}}\text{ }\text{+ }\text{λ}_{\text{1}}\text{e}_{\text{ag}\text{-}\text{sr}}\text{ }\text{+ }\text{λ}_{\text{2}}\text{e}_{\text{ag}\text{-}\text{ag}}\text{ }\text{+ }\text{λ}_{\text{3}}\text{e}_{\text{sr}\text{-}\text{sr}}$$

where $\text{λ}_{\text{1}}\text{, }\text{λ}_{\text{2}}\text{, }\text{λ}_{\text{3}}\text{ }\text{≥ 0}$ are hyperparameters that control the relative contribution of each term. We adopt the Limited-memory Broyden–Fletcher–Goldfarb–Shanno algorithm [31] to optimize the low-dimensional coordinates of antigens and antisera by minimizing the objective $\text{e}$.

Furthermore, based on the spatial distribution of antigens and antisera in the antigenic map, FluNexus provides a K-means clustering [32] functionality to facilitate the identification of meaningful clusters. Subsequently, to determine a suitable number of clusters $\text{k}$, we use the average silhouette coefficient across all samples to evaluate clustering performance for different values of $\text{k}$ and choose the value that maximizes the silhouette coefficient.

### S7 Settings of antigenic visualization

With the exception of PCA and t-SNE, all employed methods map antigens and antisera directly into a two-dimensional space. However, standard PCA and t-SNE implementations are sensitive to missing entries inherent in HI data. To address this limitation, we adopt a two-stage dimensionality reduction strategy for these two methods. First, we embed antigens and antisera into a 5-dimensional space using either FluNexus or Racmacs [2] to impute missing information. Subsequently, PCA or t-SNE is applied to map these high-dimensional embeddings into a 2-dimensional plane.

Following dimensionality reduction, k-means clustering is performed on the antigens. The number of clusters ($\text{k}$) is determined specifically for each dataset: H1 ($\text{k}\text{ = 4}$), H3 derived from Smith et al. [2] ($\text{k}\text{ = 11}$), H3 collected from WIC ($\text{k}\text{ = 13}$), and H5 ($\text{k}\text{ = 18}$). Both FluNexus and Racmacs are configured with 20,000 optimization runs and a fixed random seed of 0 to ensure reproducibility.

The fundamental principles and parameter configurations of the comparative antigenic mapping methods are detailed as follows:

- Racmacs. Racmacs employs a modified MDS algorithm to position antigens and antisera within a low-dimensional Euclidean space. The algorithm optimizes coordinates to minimize the error between map-derived distances and experimental distances. In this study, Racmacs is implemented following the protocols of Smith et al. [2] using default settings.
- UMAP. UMAP constructs a fuzzy topological representation of the dataset by approximating a manifold, assuming the data is uniformly distributed on a locally connected Riemannian manifold. The algorithm optimizes the low-dimensional embedding to minimize the fuzzy set cross-entropy between the high-dimensional and low-dimensional topological structures, thereby preserving both local and global structural features. The hyperparameters for UMAP are set as follows: n_neighbors = 15, metric = 'euclidean', init = 'spectral', and min_dist = 0.1.
- PCA. PCA reduces dimensionality by linearly mapping high-dimensional data into a lower-dimensional subspace spanned by orthogonal principal components that maximize the retained variance. In this study, PCA is implemented using *scikit-learn* with default settings, projecting data onto the first two principal components.
- t-SNE. t-SNE quantifies high-dimensional similarities as conditional probabilities and models low-dimensional embeddings using a heavy-tailed Student's t-distribution to mitigate the crowding problem. The algorithm minimizes the Kullback-Leibler divergence between these distributions to effectively preserve local manifold structures. Specifically, we employ the *scikit-learn* implementation with the following hyperparameters: perplexity = 30, learning_rate = 'auto', metric = 'euclidean', and max_iter = 1,000.

In the interactive visualization, antigenic clustering follows the PREDAC methodology using the Markov-clustering Python package with default parameters.

### S8 Quantitative metrics of antigenic map

To evaluate the accuracy of different antigenic mapping methods, we utilize MAE and MSE as performance metrics to quantify the deviation of the generated antigenic map from the original HI titer data. Following the study of Smith et al. [2], the MAE and MSE are defined as follows:

$$\text{MAE}\text{ = }\frac{\text{1}}{\text{|}\text{P}_{\text{obs}}\text{|}}\sum_{\text{(}\text{i}\text{,}\text{j}\text{) ∈ }\text{P}_{\text{obs}}} \left\{ \begin{matrix} \text{σ}\text{(}\text{D}_{\text{ij}}\text{ }\text{- }\text{d}_{\text{ij}}\text{)}\text{∙}\text{|}\text{D}_{\text{ij}}\text{ }\text{- }\text{d}_{\text{ij}}\text{|,} & \text{if}\text{ (}\text{i}\text{,}\text{j}\text{) ∈ }\text{P}_{\text{obs}}^{\text{thr}} \\ \left| \text{D}_{\text{ij}}\text{ }\text{- }\text{d}_{\text{ij}} \right|\text{,} & \text{ot}\text{h}\text{erwise} \\ & \end{matrix} \right.$$

$$\text{MSE}\text{ = }\frac{\text{1}}{\text{|}\text{P}_{\text{obs}}\text{|}}\sum_{\text{(}\text{i}\text{,}\text{j}\text{) ∈ }\text{P}_{\text{obs}}} \left\{ \begin{matrix} \text{σ}\text{(}\text{D}_{\text{ij}}\text{ }\text{- }\text{d}_{\text{ij}}\text{)}\text{∙}\text{(}\text{D}_{\text{ij}}\text{ }\text{- }\text{d}_{\text{ij}}\text{)}^{\text{2}}\text{,} & \text{if}\text{ (}\text{i}\text{,}\text{j}\text{) ∈ }\text{P}_{\text{obs}}^{\text{thr}} & \\ \left( \text{D}_{\text{ij}}\text{ }\text{- }\text{d}_{\text{ij}} \right)^{\text{2}}\text{,} & \text{ot}\text{h}\text{erwise} & \end{matrix} \right.$$

where $\text{P}_{\text{obs}}$denotes the set of antigen-antiserum pairs. $\text{P}_{\text{obs}}^{\text{t}\text{h}\text{r}}\text{ }\text{⊂ }\text{P}_{\text{obs}}$contains antigen–antiserum pairs whose HI measurements are thresholded values, typically reported as being below a detection limit (e.g., < 10). $\text{D}_{\text{ij}}$ denotes the antigenic distance between antigen $\text{i}$ and antiserum $\text{j}$ derived from experimental HI titers, while $\text{d}_{\text{ij}}$ represents the Euclidean distance between the antigen $\text{v}_{\text{i}}$ and antiserum $\text{v}_{\text{j}}$ in the constructed antigenic map. The term $\text{σ}\text{(}\text{∙}\text{)}$ denotes a sigmoid function, introduced to modulate the penalty for thresholded values.

### S9 Layout algorithm for antigenic cluster visualization

To visualize the antigenic clusters, we employ Fruchterman-Reingold force-directed algorithm, which generates a force-directed layout by modeling edges as springs that draw connected nodes together, while treating all nodes as mutually repulsive entities, analogous to anti-gravity forces. The simulation iteratively updates node positions until a near-equilibrium state is reached. This layout provides a clear representation of the network structure, facilitating the visualization of both the global architecture and local organization of the antigenic clusters.

## REFERENCES

1. Shu, Yuelong, John McCauley. 2017. “GISAID: Global initiative on sharing all influenza data–from vision to reality.” *Eurosurveillance* 22: 30494. https://doi.org/10.2807/1560-7917.ES.2017.22.13.30494
2. Smith, Derek J, Alan S Lapedes, Jan C De Jong, Theo M Bestebroer, Guus F Rimmelzwaan, Albert DME Osterhaus, Ron AM Fouchier. 2004. “Mapping the antigenic and genetic evolution of influenza virus.” *science* 305: 371–376. <https://doi.org/10.1126/science.1097211>
3. Forghani, Majid, Michael Khachay. 2020. “Convolutional neural network based approach to in silico non-anticipating prediction of antigenic distance for influenza virus.” *Viruses* 12: 1019. <https://doi.org/10.3390/v12091019>
4. Liao, Yu-Chieh, Min-Shi Lee, Chin-Yu Ko, Chao A Hsiung. 2008. “Bioinformatics models for predicting antigenic variants of influenza A/H3N2 virus.” *Bioinformatics* 24: 505–512. https://doi.org/10.1093/bioinformatics/btm638
5. Ndifon, Wilfred, Jonathan Dushoff, Simon A Levin. 2009. “On the use of hemagglutination-inhibition for influenza surveillance: surveillance data are predictive of influenza vaccine effectiveness.” *Vaccine* 27: 2447–2452. https://doi.org/10.1016/j.vaccine.2009.02.047
6. Jia, Qitao, Yuanling Xia, Fanglin Dong, Weihua Li. 2024. “MetaFluAD: meta-learning for predicting antigenic distances among influenza viruses.” *Briefings in Bioinformatics* 25: bbae395. <https://doi.org/10.1093/bib/bbae395>
7. Meng, Jing, Jingze Liu, Wenkai Song, Honglei Li, Jiangyuan Wang, Le Zhang, Yousong Peng, Aiping Wu, Taijiao Jiang. 2024. “PREDAC-CNN: predicting antigenic clusters of seasonal influenza A viruses with convolutional neural network.” *Briefings in Bioinformatics* 25: bbae033. <https://doi.org/10.1093/bib/bbae033>
8. Lee, Eva K, Haozheng Tian, Helder I Nakaya. 2020. “Antigenicity prediction and vaccine recommendation of human influenza virus A (H3N2) using convolutional neural networks.” *Human Vaccines & Immunotherapeutics* 16: 2690–2708. <https://doi.org/10.1080/21645515.2020.1734397>
9. Yin, Rui, Nyi Nyi Thwin, Pei Zhuang, Zhuoyi Lin, Chee Keong Kwoh. 2021. “IAV-CNN: a 2D convolutional neural network model to predict antigenic variants of influenza A virus.” *IEEE/ACM Transactions on Computational Biology and Bioinformatics* 19: 3497–3506. <https://doi.org/10.1109/TCBB.2021.3108971>
10. Liu, Jingze, Jiangyuan Wang, Chuan Wang, Xiao Ding, Wenping Xie, Luyao Qin, Aiping Wu, Jing Meng, Taijiao Jiang. 2025. “Deciphering the Antigenic Evolution of Seasonal Influenza A Viruses with PREDAC-Transformer: From Antigenic Clustering to Key Site Identification.” *bioRxiv* 2025.2010. 2029.684974. <https://doi.org/10.1101/2025.10.29.684974>
11. Geng, Li, Jun He, Ping Liu. 2025. “Fluattn: Antigenicity prediction of influenza a/h3n2 through attention-based feature mining.” *Infectious Disease Modelling* <https://doi.org/10.1016/j.idm.2025.11.005>
12. Du, Xiangjun, Libo Dong, Yu Lan, Yousong Peng, Aiping Wu, Ye Zhang, Weijuan Huang, Dayan Wang, Min Wang, Yuanji Guo. 2012. “Mapping of H3N2 influenza antigenic evolution in China reveals a strategy for vaccine strain recommendation.” *Nature communications* 3: 709. <https://doi.org/10.1038/ncomms1710>
13. Li, Xingyi, Yanyan Li, Xuequn Shang, Huihui Kong. 2024. “A sequence-based machine learning model for predicting antigenic distance for H3N2 influenza virus.” *Frontiers in Microbiology* 15: 1345794. <https://doi.org/10.3389/fmicb.2024.1345794>
14. Shah, Syed Awais W, Daniel P Palomar, Ian Barr, Leo LM Poon, Ahmed Abdul Quadeer, Matthew R McKay. 2024. “Seasonal antigenic prediction of influenza A H3N2 using machine learning.” *Nature communications* 15: 3833. <https://doi.org/10.1038/s41467-024-47862-9>
15. An, Yimeng, Xiao Ding, Yabin Tian, Mengyi Zhang, Yun Ma, Xi Wu, Meina Cai, Lan Huang, Yifei Wang, Jincheng Tong. 2025. “High-throughput pseudovirus neutralisation maps the antigenic landscape of influenza A/H1N1 viruses.” *EBioMedicine* 122: <https://doi.org/10.1016/j.ebiom.2025.106047>
16. Benton, Donald J, Steven J Gamblin, Peter B Rosenthal, John J Skehel. 2020. “Structural transitions in influenza haemagglutinin at membrane fusion pH.” *Nature* 583: 150–153. https://doi.org/10.1038/s41586-020-2333-6
17. Sun, Hailiang, Jialiang Yang, Tong Zhang, Li-Ping Long, Kun Jia, Guohua Yang, Richard J Webby, Xiu-Feng Wan. 2013. “Using sequence data to infer the antigenicity of influenza virus.” *MBio* 4: 10.1128/mbio.00230–00213. https://doi.org/10.1128/mbio.00230-13
18. Li, Chengjun, Masato Hatta, David F Burke, Jihui Ping, Ying Zhang, Makoto Ozawa, Andrew S Taft, Subash C Das, Anthony P Hanson, Jiasheng Song. 2016. “Selection of antigenically advanced variants of seasonal influenza viruses.” *Nature microbiology* 1: 16058. https://doi.org/10.1038/nmicrobiol.2016.58
19. Fan, Shufang, Huihui Kong, Lavanya Babujee, Robert Presler, Peter Jester, David Burke, David Pattinson, Ian Barr, Derek Smith, Gabriele Neumann. 2024. “Assessment of the antigenic evolution of a clade 6B. 1 human H1N1pdm influenza virus revealed differences between ferret and human convalescent sera.” *EBioMedicine* 101: https://doi.org/10.1016/j.ebiom.2024.105013
20. Zolotarova, Oksana, Iryna Budzanivska, Liudmyla Leibenko, Larysa Radchenko, Alla Mironenko. 2019. “Antigenic site variation in the hemagglutinin of pandemic influenza A (H1N1) pdm09 viruses between 2009–2017 in Ukraine.” *Pathogens* 8: 194. https://doi.org/10.3390/pathogens8040194
21. Koel, Björn F, David F Burke, Theo M Bestebroer, Stefan Van Der Vliet, Gerben CM Zondag, Gaby Vervaet, Eugene Skepner, Nicola S Lewis, Monique IJ Spronken, Colin A Russell. 2013. “Substitutions near the receptor binding site determine major antigenic change during influenza virus evolution.” *science* 342: 976–979. https://doi.org/10.1126/science.1244730
22. Lewis, Nicola S, Tavis K Anderson, Pravina Kitikoon, Eugene Skepner, David F Burke, Amy L Vincent. 2014. “Substitutions near the hemagglutinin receptor-binding site determine the antigenic evolution of influenza A H3N2 viruses in US swine.” *Journal of virology* 88: 4752–4763. https://doi.org/10.1128/jvi.03805-13
23. Koel, Björn F, Stefan van der Vliet, David F Burke, Theo M Bestebroer, Eny E Bharoto, I Wayan W Yasa, Inna Herliana, Brigitta M Laksono, Kemin Xu, Eugene Skepner. 2014. “Antigenic variation of clade 2.1 H5N1 virus is determined by a few amino acid substitutions immediately adjacent to the receptor binding site.” *MBio* 5: 10.1128/mbio. 01070–01014. https://doi.org/10.1128/mbio.01070-14
24. Zhang, Yuancheng, Pengfei Cui, Jianzhong Shi, Yuan Chen, Xianying Zeng, Yongping Jiang, Guobin Tian, Chengjun Li, Hualan Chen, Huihui Kong. 2023. “Key amino acid residues that determine the antigenic properties of highly pathogenic H5 influenza viruses bearing the clade 2.3. 4.4 hemagglutinin gene.” *Viruses* 15: 2249. https://doi.org/10.3390/v15112249
25. Kaufmann, Lukas, Mohammedyaseen Syedbasha, Dominik Vogt, Yvonne Hollenstein, Julia Hartmann, Janina E Linnik, Adrian Egli. 2017. “An optimized hemagglutination inhibition (HI) assay to quantify influenza-specific antibody titers.” *Journal of visualized experiments: JoVE* 55833. https://doi.org/10.3791/55833
26. Fei, MENG, MA Qi, WANG Yan, BAO Hong-mei, LIU Yan-jing, DENG Guo-hua, SHI Jian-zhong, LI Yan-bing, TIAN Guo-bin, CHEN Hua-lan. 2022. “Protective efficacy of an H5/H7 trivalent inactivated vaccine (H5-Re13, H5-Re14, and H7-Re4 strains) in chickens, ducks, and geese against newly detected H5N1, H5N6, H5N8, and H7N9 viruses.” *Journal of Integrative Agriculture* 21: 2086–2094. https://doi.org/10.1016/S2095-3119(22)63904-2
27. Influenza, WHO Global. 2011. “Manual for the laboratory diagnosis and virological surveillance of influenza.” https://ivdc.chinacdc.cn/lgzx/egl/Surveillance/sgs/201606/P020160602514925530329.pdf
28. Lapedes, Alan, Robert Farber. 2001. “The geometry of shape space: application to influenza.” *Journal of theoretical biology* 212: 57–69. https://doi.org/10.1006/jtbi.2001.2347
29. Cai, Zhipeng, Tong Zhang, Xiu-Feng Wan. 2010. “A computational framework for influenza antigenic cartography.” *PLoS computational biology* 6: e1000949. <https://doi.org/10.1371/journal.pcbi.1000949>
30. Tenenbaum, Joshua B, Vin de Silva, John C Langford. 2000. “A global geometric framework for nonlinear dimensionality reduction.” *science* 290: 2319–2323. https://doi.org/10.1126/science.290.5500.2319
31. Liu, Dong C, Jorge Nocedal. 1989. “On the limited memory BFGS method for large scale optimization.” *Mathematical programming* 45: 503–528. https://doi.org/10.1007/BF01589116
32. Hartigan, John A, Manchek A Wong. 1979. “Algorithm AS 136: A k-means clustering algorithm.” *Journal of the royal statistical society. series c (applied statistics)* 28: 100–108. https://doi.org/10.2307/2346830

## Supplementary Figures


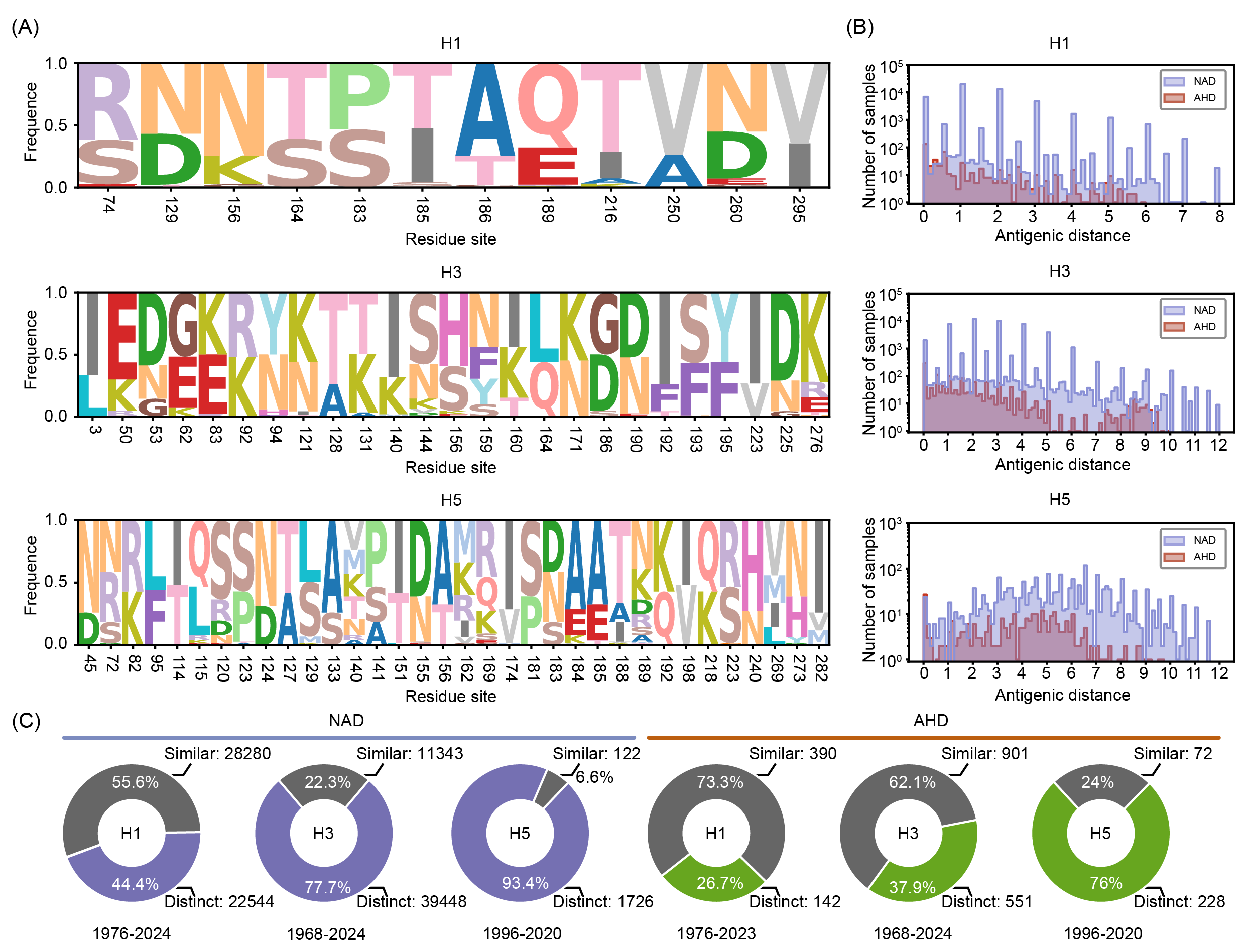


**Figure S1 Overview of hemagglutination inhibition (HI) and hemagglutinin subunit 1 (HA1) datasets for H1, H3, and H5 influenza A subtypes.** (A) Sequence logo plots of mutation hotspot regions within HA1 for each subtype. (B) Distribution of antigenic distances. (C) Distribution of the number of antigenically similar and distinct samples.

**Figure S2 Performance evaluation of methods using the Normalized Antigenic Distance (NAD) antigenic distance.** (A) Performance of discriminating antigenic variants from non-variants. (B) Performance of inferring antigenic distance. (C) Comparison of feature encoding and training time across methods for H3 subtype. (D) Summary of method performance. The bars represent the relative rankings of model performance, with longer bars indicating better performance.

**Figure S3 Performance evaluation of methods using the log2-transformed Archetti–Horsfall Distance (AHD) antigenic distance.** (A) Performance of different methods in distinguishing antigenic variants from non-variants. (B) Performance of different methods in inferring antigenic distance.

**Figure S4 Comparison of feature encoding and training time across methods.** (A) Feature encoding and training time across methods for H1 subtype using the NAD antigenic distance. (B) Feature encoding and training time across methods for H5 subtype using the NAD antigenic distance. (C) Feature encoding and training time across methods for H3 subtype using the AHD antigenic distance. (D) Feature encoding and training time across methods for H1 subtype using the AHD antigenic distance. (E) Feature encoding and training time across methods for H5 subtype using the AHD antigenic distance.

**Figure S5 Overview of method performance.** (A) Summary of method performance for the NAD distance metric under retrospective testing. (B) Summary of method performance for the AHD distance metric under cross-validation. (C) Summary of method performance for the AHD distance metric under retrospective testing.

**Figure S6 Antigenic maps generated by FluNexus, Racmacs, Uniform Manifold Approximation and Projection (UMAP), principal component analysis (PCA), and t-distributed Stochastic Neighbor Embedding (t-SNE) using the HI data of H3 subtype derived from the annual and interim reports of the Worldwide Influenza Centre at the Francis Crick Institute under temporal sampling.** For each method, antigens are colored consistently with the antigenic map generated from their complete HI data, with distinct colors corresponding to the antigenic clusters predicted by the respective method.

**Figure S7 Antigenic maps generated by FluNexus, Racmacs, UMAP, PCA, and t-SNE using the HI data of H1 subtype under temporal sampling.** For each method, antigens are colored consistently with the antigenic map generated from their complete HI data, with distinct colors corresponding to the antigenic clusters predicted by the respective method.

**Figure S8 Antigenic maps generated by FluNexus, Racmacs, UMAP, PCA, and t-SNE using the HI data of H5 subtype under temporal sampling.** For each method, antigens are colored consistently with the antigenic map generated from their complete HI data, with distinct colors corresponding to the antigenic clusters predicted by the respective method.
